# Malaria Parasitaemia among Infants and Its Association with Breastfeeding Peer Counselling and Vitamin A Supplementation: A Secondary Analysis of a Cluster Randomized Trial

**DOI:** 10.1371/journal.pone.0021862

**Published:** 2011-07-07

**Authors:** Victoria Nankabirwa, Thorkild Tylleskar, Jolly Nankunda, Ingunn Marie S. Engebretsen, Halvor Sommerfelt, James K. Tumwine, for the PROMISE EBF Research Consortium

**Affiliations:** 1 Department of Paediatrics and Child Health, School of Medicine, College of Health Sciences, Makerere University, Kampala, Uganda; 2 Centre for International Health, University of Bergen, Bergen, Norway; 3 Division of Infectious Disease Control, Norwegian Institute of Public Health, Oslo, Norway; The George Washington University Medical Center, United States of America

## Abstract

**Background:**

Malaria is the second highest contributor to the disease burden in Africa and there is a need to identify low cost prevention strategies. The objectives of this study were to estimate the prevalence of malaria parasitaemia among infants and to measure the association between peer counselling for exclusive breastfeeding (EBF), vitamin A supplementation, anthropometric status (weight and length) and malaria parasitaemia.

**Methods:**

A cluster randomized intervention trial was conducted between 2006 and 2008 where 12 of 24 clusters, each comprising one or two villages, in Eastern Uganda were allocated to receive peer counselling for EBF. Women in their third trimester of pregnancy (based on the last normal menstrual period) were recruited in all 24 clusters and followed up until their children's first birthday. Blood was drawn from 483 infants between 3 and 12 months of age, to test for malaria parasitaemia.

**Results:**

The prevalence of malaria parasitaemia was 11% in the intervention areas and 10% in the control areas. The intervention did not seem to decrease the prevalence of malaria (PR 1.7; 95% CI: 0.9, 3.3). After controlling for potential confounders, infants not supplemented with Vitamin A had a higher prevalence for malaria compared to those who had been supplemented (PR 6.1; 95% CI: 2.1, 17.6). Among children supplemented with vitamin A, every unit increase in length-for-age Z (LAZ) scores was associated with a reduced prevalence in malaria (PR 0.5; 95% CI:0.4, 0.6). There was no association between LAZ scores and malaria among children that had not been supplemented.

**Conclusion:**

Peer counselling for exclusive breastfeeding did not decrease the prevalence of malaria parasitaemia. Children that had not received Vitamin A supplementation had a higher prevalence of malaria compared to children that had been supplemented.

**Trial registration:**

Clinicaltrials.gov: NCT00397150.

## Introduction

Malaria was ranked in 2004 as the eighth highest contributor to the global disease burden and the second highest contributor in Africa. It was responsible for 2.9% of the global disability-adjusted life years (DALYs) lost and 10.1% of the total DALYs lost in Africa [1]. Though the *plasmodium* species were discovered over 100 years ago, and the female anopheles mosquito identified as the vector, malaria continues to be the most significant parasitic disease among humans [2]. Presently, it accounts for about 500 million cases each year and approximately 1 to 3 million deaths [2], [3], [4]. In Uganda, it has been the most important cause of illness and death for decades [5]. The failed attempt by WHO to eradicate malaria in the fifties, coupled with weak control programmes and the emergence of anti-malarial drug resistance has resulted in a persistence of malaria in Sub-Saharan Africa, Southeast Asia and South America [6].

Populations in these malaria endemic areas often live under conditions that predispose to malnutrition. Moreover, children, who have the highest risk of severe malaria often have the highest risk of poor nutrition [4]. But the association between malaria and nutrition is complex and studies are inconsistent [7]. In the seventies s, observational studies indicated that malnutrition may protect children from malaria [8], [9]. Newer studies have shown either no effect or a protective effect of adequate nutrition [3], [10], [11]. Exclusive breastfeeding (EBF) in the first months of life has been shown to reduce diarrhoea morbidity [12], but the association between EBF and malaria is unclear. A few studies have reported a reduced risk of malaria among exclusively breastfed infants but other studies have found no such effect [13], [14]. Similar debate surrounds the association between vitamin A supplementation and malaria [15], [16]. Randomised controlled trials in Burkina Faso and Papua New Guinea found a significant decrease in the prevalence and episodes of malaria in the intervention group [15], [17]. The Burkina study also found a longer time period to first malaria episode in the intervention group [17]. On the other hand, studies in Tanzania and Ghana found no association between vitamin A supplementation and malaria [16], [18]. The safety of vitamin A supplementation has also been questioned. Studies in Guinea-Bissau found that girls who received vitamin A at birth had a two-fold higher mortality when they received diphtheria – tetanus – pertussis (DPT) [19], [20]. These findings were corroborated by a study in animal models that also showed higher levels of parasitaemia in mice supplemented with both vitamin A and DPT [21]. But, a systematic review of vitamin A supplementation for postpartum women found no adverse events attributable to vitamin A supplementation [22]. This general lack of consensus highlights the gaps in the malaria-nutrition literature. In Uganda, the national expanded programme on immunization recommends that children between six and twelve months of age receive 100,000 IU of vitamin A while children less than six months whose mothers' did not receive postpartum vitamin A supplementation or who are not breastfed receive 50,000 IU. The objectives of this study were:1) to estimate the prevalence of malaria parasitaemia among infants, 2) to measure the association between peer counselling for exclusive breastfeeding and prevalence of malaria and 3) to measure the effect of vitamin A supplementation and anthropometric status (weight and length) on the prevalence of malaria.

## Methods

The protocol for this trial and supporting CONSORT checklist are available as supporting information; see Checklist S1 and Protocol S1.

### Ethics statement

We obtained written informed consent from each study participant. Ethical approval was obtained from the Makerere University Research and Ethics Committee, the Uganda National Council for Science and Technology, and from the Regional Committee for Medical and Research Ethics for Western Norway (REK VEST, approval number 05/8197).

This was a sub-study undertaken during a cluster-randomized behaviour intervention trial (the PROMISE EBF trial), in which exclusive breastfeeding for the first six months of life by individual peer counselling in the intervention areas was promoted (Clinical trials gov: NCT00397150) [23]. To assess the potential for scaling up this programme-relevant intervention, clusters rather than individual mother-infant pairs were randomized, thereby avoiding the potential spill-over between intervention and control arms. Randomisation was stratified by urban/rural populations. Clusters were mapped based on criteria of accessibility, population size, health system and health statistics then randomised by the central coordinating team into intervention or control arms. Intervention clusters received breastfeeding support from peer counsellors while control clusters received standard health care only. Data collection was carried out between 2006 and 2008.

### Study site

The study was conducted in Mbale district which had an estimated population of 720,000 [24] and is located 300 km North-East of Kampala. Mbale district, lying at the foothills of Mt Elgon is a high altitude area lying between 1200 m and 2100 m. Average annual rainfall is 1500 mm^2^. There are two rain seasons: mid-February to end of May and August to December. Malaria is holoendemic in this area, mostly caused by *plasmodium falciparum*. The area is served by Mbale hospital, which doubles as the district and regional referral hospital. HIV prevalence among pregnant women in antenatal clinics in Mbale was approximately 5–6% during the study period. Most of the people living in the area were subsistence farmers.

Mbale district comprised of seven counties; the study was conducted in the two biggest counties, namely Bungokho County (rural) and Mbale Municipality (urban). Twenty four clusters were included in the study, 18 rural and 6 urban. The six urban clusters in Mbale municipality were selected from all its three municipal divisions. Most of the urban areas were large informal settlements. Eighteen clusters in Bungokho County were chosen from eight of its eleven sub-counties. Clusters were included if they neighboured the main road out from Mbale Municipality or were on the 1^st^ or 2^nd^ branch off the main road, had a population of at least 1,000 inhabitants and represented a social and administrative unit. Efforts were made to avoid contamination between clusters. Twelve clusters were randomly designated to receive the intervention and 12 were left as control clusters.

### Sample size and Study subjects

**Figure 1:**
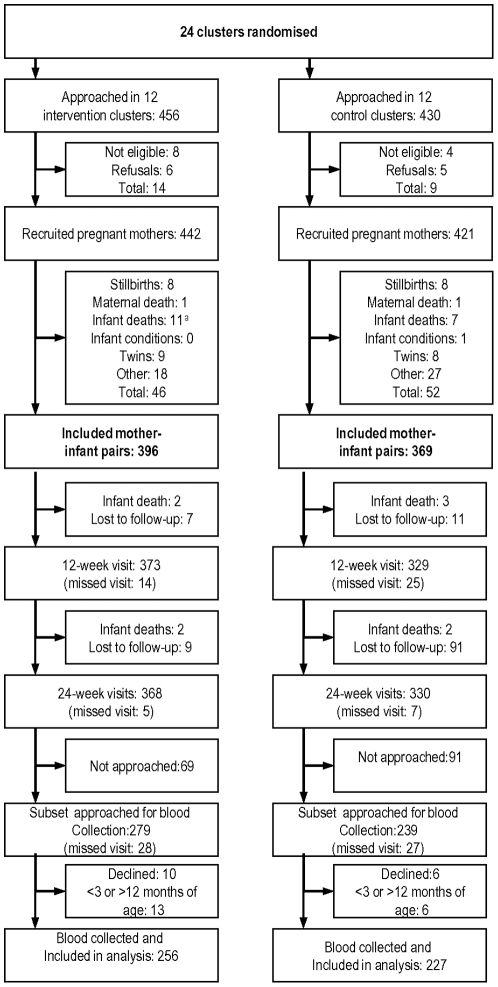
Flow of study participants.

Assuming a parasitaemia prevalence of 50% in the control group, an alpha error of 0.05, and power of 80%, a total sample size of 368 infants was required to find a prevalence ratio of 0.7. In the PROMISE EBF trial all pregnant women in the selected clusters were approached by the study team. They were eligible if they resided in the study area, were at least seven months pregnant (based on the last normal menstrual period) or visibly pregnant, opted to breastfeed their infants and consented to participate in the study. In addition, women were excluded if they had an intention to leave the area during the study period, gave birth to twins or to infants with serious illnesses or deformities like cleft lip/palate that could interfere with breastfeeding. A total of 886 pregnant women were identified and approached. Of these, 875 women (99%) accepted to participate in the study. Out of the 875 women, 12 (1%) did not meet the eligibility criteria and 28 (3%) relocated out of the study area after recruitment but before delivery and were lost to follow-up. There were 835 deliveries. These children were followed up and blood was drawn from a subsample of 483 infants between 3 and 12 months. Children were enrolled into this sub sample consecutively, starting with the oldest until a sufficient sample size was attained. Children older than one year at the time of sample collection were excluded from the study. Because of the cluster design of the Promise-EBF study, once a cluster was visited, all eligible children were approached during the visit. All clusters were visited a couple of times until an adequate sample size was achieved. Figure 1 shows the flow of study participants. Analysis for this study is based on this subsample.

### Intervention

The intervention was peer counselling for exclusive breastfeeding in the first six months of life. Peer counsellors were identified from the local community and with the help of the local community. They attended a one week training course in which simplified materials based on the WHO breastfeeding counselling course were used [25]. Peer counselling for breastfeeding was done during home visits (arranged at the convenience of the time of day) to the mother. Each mother was visited at least five times, once during pregnancy, and then in the first, fourth, seventh and tenth week after birth. Peer counsellors provided substantial information, encouragement, skills and support for achieving exclusive breastfeeding, if possible for six months.

### The control arm

The control clusters received only the routine government breastfeeding counselling services available at the time and this was mainly counselling for exclusive breastfeeding during antenatal care visits at the health centres.

### Exposures

Residence in the intervention clusters, vitamin A supplementation and nutritional status were the main exposures in this study. Pre-tested structured questionnaires in the local language (Lumasaaba) were used to collect information on these exposures as well as on potential confounders such as socio-demographic characteristics, current pregnancy, immunization status, water and sanitation, previous pregnancies (parity and child survival) and use of mosquito bed nets. Current breastfeeding was assessed at 3, 6, 12 and 24 week postpartum visits using a 24-hour and last 7-day recall. In addition, dietary recalls included 22 specified items and one open option. Breastfeeding mothers who did not give any other food or liquids other than breast milk disregarding medicines were categorised as exclusively breastfed, even if their infant had received medications [26]. Weight was measured at 12 weeks using standardized Salter spring scales to the nearest 0.1 kg. The scales were calibrated before each field visit. Length was measured at 12 weeks using standard length boards to the nearest millimeter. Receipt of vitamin A was based on information drawn from the child health immunization card. We also had data based on maternal recall of receipt of vitamin A, but we regard the data from the health cards to be of better quality and thus used this data as far as it was available. Data collectors were recruited from the study area and were fluent in the local language. They were periodically trained on the questionnaire and anthropometry. Rainfall data for the period between 2007 and 2008 for the area was obtained from the ministry of agriculture animal industry and fisheries records for Buginyanya research station.

### Outcome

The outcome measure in this study was malaria parasitaemia. A blood sample for parasitaemia (thick and thin blood film) was collected from a finger prick by a trained laboratory technologist from all children in the sub sample. Blood slides were air dried and the thin smears were fixed in 99% methanol. The slides were stained with 10% Giemsa solution for 10 minutes. The number of asexual *P.falciparum* malaria parasites per 200 white blood cells was determined on the thick film by a trained laboratory technologist who was blinded to the intervention allocation, vitamin A status and anthropometric values. Thin smears were used to identify the parasite species while thick smears were used to estimate parasite densities. Parasite density per microlitre (µl) was calculated assuming a white blood count of 8×10^3^/µl.

### Blinding (masking)

Data collectors and the laboratory technologist were blinded to the random allocation of the clusters. However, this being a behaviour change community trial, it was difficult to fully blind the data collectors and participants.

### Statistical methods

Data was directly entered into hand held computers in the field using EpiHandy software (www.openXdata.org, version 165.528-142 RC). Data analysis was undertaken using Stata version 9 (StataCorp LP, TX, U.S.). Exposures were residence in an intervention cluster, exclusive breastfeeding, vitamin A supplementation and nutritional status. Weight-for-length (WLZ), length-for-age (LAZ) and weight-for-age (WAZ) Z-scores were calculated with the software WHO Anthro for personal computers, version 2 [27]. Children were classified as wasted, stunted and underweight if they had WLZ<−2, LAZ<−2 and WAZ<−2, respectively. The outcome, malaria was analyzed as a binary outcome. We did have quantitative data on parasitaemia density. But an analysis of malaria as a continuous outcome yielded results similar to those obtained with malaria as a binary variable. This is because those without malaria had a zero count and there was not much variation in the count for those with malaria.

We created a composite index of wealth (socio-economic status) using multiple correspondence analysis (MCA). We used MCA on possession of a TV, radio, mobile phone, chair, cupboard, refrigerator, type of toilet, type of house walls as well as presence of electricity and water in the home.

Crude prevalence ratios (PR) and prevalence differences (PD) and 95% confidence intervals were estimated for the exposure variables. We used multivariable generalized linear model (GLM) regression analysis with a log link to estimate the adjusted PR of the independent variables (exposures) on malaria parasitaemia. All final estimates were adjusted for bed net use, age of the infant, wealth, residence and the design effect. We tested for effect measure modification (interaction) on both additive and multiplicative scales between vitamin A supplementation and anthropometric status. Cross product terms were used to test for interaction on the multiplicative scale.

## Results

Blood samples were collected from a total of 483 infants. The mean age at blood collection was 6.5 months (S.D = 1.7, range 3–12 months). Half of the infants (242) were girls. The mean age of the mothers was 25 years, (range 14 to 44 years) and they had an average of 6 years of formal education (range 0 to 16 years). At 24 weeks, 205 (42%) of the children had received vitamin A supplementation. There were no baseline differences between the infants and mothers of infants in the intervention and control clusters in terms of age and sex of the children, mother's age, education, religion, or parity (table 1 and 2). Bed net use was 42% (108 out of 256) in the intervention arm and 50% (113 out of 227) in the control group (table 2).

**Table 1:** Basic socio-demographic characteristics of mothers in the intervention and control groups.

Characteristic	Intervention group (n = 256)	Control group (n = 227)
Mother's age		
≤19	58 (23%)	44 (19%)
20–24	79 (31%)	67 (30%)
25–29	60 (23%)	54 (24%)
≥30	59 (23%)	62 (27%)
Mother's Education		
None	20 (8%)	30 (13%)
1–4 years	48 (19%)	45 (20%)
5–7 years	121 (47%)	83 (37%)
8–11 years	60 (23%)	57 (25%)
12 or more years	7 (3%)	12 (5%)
Residence		
Urban	57 (22%)	34 (15%)
Rural	199 (78%)	193 (85%)
Marital Status		
Married	167 (65%)	152 (67%)
Cohabiting	72 (28%)	58 (26%)
Other*	17 (7%)	17 (7%)
Religion		
Catholic	49 (19%)	41 (18%)
Protestant	112 (44%)	98 (43%)
Islam	87 (34%)	79 (35%)
Other	8 (3%)	9 (4%)
Household wealth index		
Poorest 20%	68 (27%)	39 (17%)
Middle 40%	113 (44%)	88 (39%)
Richest 40%	75 (29%)	100 (44%)
Antenatal care attendance		
No	80 (31%)	49 (22%)
Yes	176 (69%)	178 (78%)

*Includes single, widowed, divorced, separated.

**Table 2:** Basic socio-demographic characteristics of infants in the intervention and control groups.

Characteristic	Intervention group (n = 256)	Control group (n = 227)
Number of siblings		
0	54 (21%)	53 (23%)
1	36 (14%)	29 (13%)
2	34 (13%)	33 (15%)
3	33 (13%)	26 (11%)
> = 4	99 (39%)	86 (38%)
Gender of infant		
Girl	133 (52%)	109 (48%)
Boy	123 (48%)	118 (52%)
Place of delivery		
Home	126 (49%)	84 (37%)
Hospital/Local maternity/clinic/TBA/other	130 (51%)	143 (63%)
Vitamin A supplementation		
No	151 (59%)	127 (56%)
Yes	105 (41%)	100 (44%)
Age of infant		
3–6 months	142 (55%)	135 (58%)
7–12 months	114 (45%)	95 (42%)
Use of Mosquito net		
Yes	108 (42%)	113 (50%)
No	148 (58%)	114 (50%)

### Malaria and rainfall

Prevalence of *P. falciparum* malaria was 11% (51 out of 483 children). Malaria prevalence was highest in October (3/22, 14%) which was also the month that registered the highest amount of rainfall (928.6 mm). In the period between July and October 2007, we registered the highest amounts of rainfall and the highest prevalence of malaria parasitaemia. The period between November 2007 and March 2008 had the lowest amount of rainfall and the lowest prevalence of parasitaemia.

### Malaria and breastfeeding, residence, use of bed nets, and education

Prevalence of *P. falciparum* malaria was 11% in the intervention area and 10% in the control areas (table 3). The intervention did not seem to decrease the prevalence of malaria (PR 1.7; 95% CI: 0.9, 3.3).

**Table 3:** Malaria and the peer counselling for breastfeeding intervention in a cohort of 483 infants in Mbale, Eastern Uganda^a^.

Characteristic	N (%) n = 483	Malaria cases (prevalence) n = 51	PR (unadjusted) 95% CI	PR (adjusted)^b^ 95% CI
Allocation				
Intervention	256 (53%)	29 (11%)	1.2 (0.7, 2.0)	1.7 (0.9, 3.3)
control	227 (47%)	22 (10%)		
Use of bed nets				
No	262 (54%)	39 (15%)	2.7 (1.5, 5.1)	2.2 (1.0, 5.1)
Yes	221 (46%)	12 (5%)		
Age of infant				
3–6 months	274 (57%)	23 (8%)		
7–12 months	209 (43%)	28 (13%)	1.6 (0.9, 2.7)	1.4 (0.7, 2.6)
Residence				
Rural	425 (81%)	56 (13%)	11.6 (1.6, 82.9)	10.0(1.5, 66.3)
Urban	98 (19%)	1 (1%)		
Household wealth index				
Poorest 20%	107 (22%)	15 (14%)		
Middle 40%	201 (42%)	24 (12%)	0.9 (0.5, 1.6)	1.2 (0.6, 2.4)
Richest 40%	175 (36%)	12 (7%)	0.5 (0.2, 1.0)	1.2 (0.4, 3.2)
Place of delivery				
Home	210 (43%)	21 (10%)	1.1 (0.6, 1.9)	
Hospital/Local maternity/clinic/TBA/other	273 (57%)	30 (11%)		
Antenatal care attendance				
No	129 (27%)	14 (11%)	1.0 (0.6, 1.9)	
Yes	354 (73%)	37 (10%)		

a *PR indicates prevalence ratio, CI, confidence interval*.

b *Model adjusted for season and cluster*.

All the children included in the study were breastfed. There was no association between exclusive breastfeeding in the first six months of life and malaria (prevalence ratio 1.3; 95% CI: 0.7, 2.2). At 12 weeks, infants in intervention areas were more likely to be exclusively breastfed than infants in control areas (prevalence ratio 1.89; 95%CI 1.70, 2.11). Detailed results on the effect of the peer counselling intervention on breastfeeding have been presented elsewhere [23].

In a crude analysis, children in rural areas were 12 times more likely to have malaria than were children in the urban areas. After adjusting for use of bed nets, age of the child and intervention allocation, children in rural areas were still more likely to have malaria than children in urban areas (PR 9.8; 95% CI:1.4, 67.1). Infants whose mothers did not use bed nets were more likely to have malaria compared to those whose mothers' used bed nets. There was no association between maternal education, gender, antenatal care attendance or place of delivery and malaria (table 3).

### Malaria and vitamin A supplementation and anthropometry

Out of 205 children that had received Vitamin A supplementation according to the vaccination cards, 11 (5%) had positive blood slides for *P. falciparum* compared to 14% (40 out of 278) of the children that had not received Vitamin A supplementation. In a crude analysis, children who had not received vitamin A were more likely to have malaria parasitaemia than those who had received it (PR 2.7; 95% CI: 1.4, 5.1). The association with vitamin A strengthened after controlling for use of bed nets, age and residence (PR 6.1; 95% CI: 2.1, 17.6) (table 4).

**Table 4:** Association between vitamin A supplementation, anthropometric status and malaria among infants in Mbale, Eastern Uganda^a^.

Characteristic	PR (unadjusted)	PR (adjusted)^b^	PR (adjusted)^c^	PR (adjusted)^d^	PR (adjusted)^e^
	95% CI	95% CI	95% CI	95% CI	95% CI
Vitamin A Supplementation					
No	2.7 (1.4, 5.1)	5.2 (1.9, 14.8)	6.1 (2.1, 17.6)	12.3 (3.8, 40.1)	15.7 (4.2, 58.9)
Yes	1	1	1	1	1
LAZ	0.9 (0.7, 1.2)	2.6 (0.3, 19.8)	0.8 (0.1, 8.0)	1.0 (0.1, 8.5)	0.4 (0.0, 2.9)
WLZ	1.0 (0.8, 1.2)	2.6 (0.4, 15.8)	0.9 (0.1,7.1)	2.7 (0.5, 15.8)	1.3 (0.2, 7.5)
WAZ	0.9 (0.7, 1.1)	0.2 (0.0, 3.3)	0.9 (0.1,17.2)	0.4 (0.0, 4.9)	1. 2 (0.1, 13.9)
Vitamin A supplementation * LAZ				3.1 (1.6, 6.2)	3.7 (1.8, 7.7)
Vitamin A supplementation * WLZ				0.5 (0.3, 1.1)	0.5 (0.2, 0.9)

a *Analysis restricted to 380 infants with complete anthropometric data, PR indicates prevalence ratio, CI, confidence interval, LAZ, length for age Z scores, WLZ, weight for length Z scores, WAZ, weight for age Z scores*.

b *Model adjusted for bed nets, age, residence and cluster*.

c *Model adjusted for bed nets, age, residence, cluster, wealth and season*.

d *Model adjusted for bed nets, age, residence, wealth and cluster*.

e *Model adjusted for bed nets, age, residence, wealth, cluster and season*.

The mean weight for length Z score (WLZ) was 0.12±1.3, inter-quartile range (IQR): −0.81, 1.0. Mean length for age Z score (LAZ) was −0.7±1.1 (IQR: −1.5 to 0.1) and the mean weight for age (WAZ) was −0.5±1.2 (IQR: −1.2 to 0.2) at 12 weeks. Further, 7% of the children were wasted, 11% were stunted and 9% were underweight. There was no association between length for age Z scores (LAZ) and malaria parasitaemia. However, there was effect measure modification on both the additive and multiplicative scales between LAZ and vitamin A supplementation as indicated by a significant cross product term (table 4). Among children who had been supplemented with vitamin A, every unit increase in LAZ score was associated a 50% reduced prevalence of malaria parasitaemia (PR 0.5; 95% CI: 0.4, 0.6). Increments in LAZ scores were not associated with a reduced prevalence of malaria parasitaemia among children who had not been supplemented with vitamin A (PR 1.0; 95% CI: 0.7, 1.4). P-value for cross product term was <0.001.

The excess prevalence of malaria attributable to stunting was substantially greater among those who had not been supplemented with Vitamin A; prevalence difference 38/1,000 (95%CI: 8/1,000, 68/1,000) than among those children who had received Vitamin A supplementation; prevalence difference −6/1,000 (95%CI −158/1,000, 146/1,000), P-value for homogeneity of prevalence differences = 0.013.

## Discussion

Though the relationship between malaria and nutrition is complex, increasingly, evidence suggests an association between the two [3], [4]. This study estimated the prevalence of malaria parasitaemia among infants and assessed the effect of peer counselling for exclusive breastfeeding, vitamin A supplementation and anthropometric status on the prevalence of malaria parasitaemia.

In this study, peer counselling for exclusive breastfeeding did not decrease the prevalence of malaria parasitaemia. Similarly, exclusive breastfeeding for the same period did not have a significant impact on the prevalence of malaria parasitaemia. It has been hypothesized that the possible protective effect of breastfeeding is through immuno-protective mechanisms or alternately related to lactoferrin. Lactoferrin is a protein found in breast milk and neutrophils, with antibacterial properties as well as lipoprotein remnants [28]. Its protective effect is postulated to be through inhibition of the invasion of hepatocytes by malarial sporozoites, because they compete for the same receptor in the hepatocyte plasma membrane [28]. A cross-sectional study among Nigerian children found a significantly higher prevalence of malaria parasitaemia among exclusively breastfed children [14]. Conversely, a study among Malawian children found that children who were not exclusively breastfed had a marginally increased prevalence of malaria episodes [13].

Children who had not received Vitamin A supplements were about six times more likely to have *P. falciparum* malaria than those who had received them. In Uganda, the national expanded programme on immunization recommends that children between six and twelve months of age receive 100,000 IU of vitamin A while children less than six months whose mothers' did not receive postpartum vitamin A supplementation or who are not breastfeeding receive 50,000 IU. Vitamin A is necessary for normal immune function and although earlier animal studies showed vitamin A deficiency to be protective, it is now hypothesized that vitamin A could protect against malaria. Cross-sectional studies in humans have shown vitamin A to be associated with malaria, but causality has been uncertain [29]. To date, there are only a few prospective studies that have studied the relationship between vitamin A and malaria. A double blind placebo-controlled trial in Papua New Guinea reported that vitamin A supplementation reduced the frequency of *P. falciparum* malaria by 30% among preschool children [15]. A second clinical trial in Ghana found no association between vitamin A supplementation and morbidity due to malaria [16]. However, the Ghanian study did not have sufficient power to detect a difference of less than 70% between the two groups [30]. The effect of vitamin A supplementation in this study is in line with findings from the Papua New Guinea study and the Burkina Faso study [17]. It is hypothesized that Vitamin A acts by increasing phagocytosis of parasitized erythrocytes as well as reducing the proinflammatory response to the malaria infection [31]. This is because vitamin A may assist in the up-regulation of CD36 expression which facilitates phagocytosis.

Though the weight for length Z scores were in line with the WHO growth reference standards, the prevalence of stunting (length for age Z score<−2) was quite high (11%) at 12 weeks. There was no association between nutritional status and prevalence of malaria parasitaemia. This is in line with findings from the Democratic Republic of Congo [11], where a cross-sectional study among children found no association between *plasmodium* infection and nutritional indicators. However, a Gambian case control study among children admitted in hospital found mean admission weights to be lower for children admitted with malaria than for controls [10]. The case control design of this Gambian study leaves causality and temporality uncertain. A recent study in Papua New Guinea found that there was no association between wasting and malaria but that stunting was protective [7]. Though recent literature has found no evidence of nutritional modulation of malaria or a protective effect of good nutrition, earlier studies including animal studies, clinic based studies and studies from re-feeding programmes pointed to a protective effect of malnutrition [8], [9]. Careful analysis of these studies reveals that often, nutritional status was based on qualitative and subjective indicators unlike today where reference is made to WHO standards.

Children living in rural areas were more likely to have malaria parasitaemia than those in urban areas. This is comparable to national statistics that show a higher prevalence in rural areas [5]. This is probably because there are more vector breeding sites in rural areas. Public health malaria prevention programmes would do well to target these areas. Months with the highest rainfall were also characterized by high malaria prevalence estimates. This is similar to other findings in Uganda and elsewhere [32], [33]. Because of the El Niño/Southern Oscillation (ENSO) related climate anomalies it is predicted that the East African region will have more abnormal and heavier rainfall. We can therefore expect higher malaria prevalence unless adequate malaria prevention programmes are in place.

The strength of this study lies in its prospective nature. In addition, data was collected from children in the community which enabled us to obtain malaria prevalence and make comparisons from the general population rather than from hospital cases as is often the case. Generalizability in this study is limited by the fact that clusters included in this study were close to main roads. We believe these findings can be applicable to regions with similar socio-demographic characteristics. Diagnosis of malaria in this study was based on microscopy. Microscopy has been shown to have a sensitivity of 30.5% and a specificity of 100% in comparison to PCR [34]. Despite the fact that the microscopist in this study was well trained, the low sensitivity of microscopy may have led misclassification of true malaria cases as non-cases, possibly biasing the observed measures of effect towards the null. We therefore, would expect the true measures of effect to be larger than those observed in this study.

In conclusion, literature on the relationship between malaria and nutrition is controversial. This study shows no protective effect of peer counselling for exclusive breastfeeding against malaria. Children that had not received Vitamin A supplementation had a higher prevalence of malaria compared to children that had been supplemented. Among children supplemented with vitamin A, every unit increase in LAZ scores was associated with a reduced prevalence in malaria. There was no association between LAZ scores and malaria among children that had not been supplemented. We recommend further studies to explore the role of vitamin A supplementation in malaria endemic areas as an adjunct malaria prevention strategy.

## Supporting Information

Protocol S1 **The PROMISE- EBF protocol and amendment of the protocol to include prevalence of malaria as one of the outcome measures.**
(DOC)

Checklist S1 **CONSORT 2010 checklist of information to include when reporting a randomised trial.**
(DOC)
