# Supplementary material for: Malaria Parasitaemia among Infants and Its Association with Breastfeeding Peer Counselling and Vitamin A Supplementation: A Secondary Analysis of a Cluster Randomized Trial
Source: PLoS One. 2011 Jul 7;6(7):e21862. doi: 10.1371/journal.pone.0021862 (PMC3131393; doi:10.1371/journal.pone.0021862)
Supplement: Protocol S1 — The PROMISE- EBF protocol and amendment of the protocol to include prevalence of malaria as one of the outcome measures. (DOC) [file pone.0021862.s001.doc]

The PROMISE- EBF protocol and amendment of the protocol to include prevalence of malaria as one of the outcome measures

1. The PROMISE-EBF protocol

***Abstract***

**Introduction**: promotion of EBF is one of the most effective child health interventions currently feasible for implementation at population level in low income countries. It can lower infant mortality by 13%. EBF is little appreciated by mothers and as a result EBF rates are very low in Africa. Studies have shown that peer counselling can achieve dramatic increases in EBF rates. Thus we propose to run a randomized trial to test this model in Mbale District, Eastern Uganda and to quantify health benefits and implications for the health care system.

**Objectives**: to assess the effect of peer counselling on improving EBF prevalence at 3 months, in Mbale District Eastern Uganda.

**Design**: a Randomized Control Trial testing the efficacy and safety of promotion of EBF. Randomization will be done at community level to peer counselling or no peer counselling.

**Method**: the .study will be conducted in Mbale Municipality and Bunghoko County. Twenty four clusters will be identified and randomised to either peer counselling or no peer counselling (12 clusters in each arm). Consenting pregnant mothers less than 40 years old, with intention to breast feed their babies will be recruited in the study area.

Recruitment will be done in the late ante-natal period to establish eligibility. Eligible mothers living in clusters randomized to peer counselling will receive individual contact with a peer counsellor. Mothers in the non-intervention clusters will receive the standard health education messages from the Ministry of Health. Both groups will be followed up at 3, 6, 12 and 24 weeks post partum to determine infant feeding patterns, morbidity, survival and, anthropometric measurements of infants. Blood samples will be taken from the infants at 24 weeks to determine their micro-nutrient status.

**Data collection and analysis**: basic data will be collected on all participants and the primary outcome measure will be EBF prevalence at 3 months post-natally. Data will be collected on questionnaires on hand-held computers. Data will be automatically uploaded from the hand-held to the site data base. Analysis will be by intention to treat.

**Utility*:*** the results of the study will be used to develop a safe and effective means of promoting EBF taking into account the need to minimize postnatal HIV transmission

***Introduction***

More than 10 million children die each year from preventable causes and almost 90% from poor countries (Black RE et aI, 2003). About 41 % of child deaths occur in sub-Saharan Africa from diarrhoea, pneumonia, malaria and HIV/AIDS; with under-nutrition as an underlying cause of two- fifths of all child death (UNICEF, 2003). Since 2000, when the United Nations Millennium Declaration was signed, efforts to reduce mortality among children younger than five years of age have been accelerating. It will be difficult to reach the stated goal - cutting the rate by two thirds by 2015(UN, 2001) - without reducing the number of infant deaths. Many useful interventions can be implemented in resource poor settings, but weak health care delivery systems remain an important barrier. The under-5 mortality in Uganda is estimated at 140 deaths per 1000 live births (UDHS, 2000-2001).About two-thirds of child deaths could be prevented by interventions that are available today and are feasible for implementation in low-income countries at high levels of population coverage. One of the most effective of these is the promotion of exclusive breastfeeding (EBF), capable of lowering child deaths by 13%(Black RE et aI, 2003) which means it could save some 15 million children from dying over the next 10 years.

The benefits of breastfeeding and the negative effects of artificial feeding in underprivileged environments were brought to public attention in the 1970s. A decade of research in the 1970s confirmed that breastfed babies do not need anything beside breast milk, not even water in hot climates (Almroth SG, 1978). In the early 1990s, UNICEF started the "Baby-friendly Hospital Initiative" (BFHI) which promoted breastfeeding and resulted in significant reduction in child morbidity and mortality. The Demographic and Health Survey (DHS) for Uganda 2000-2001 stated that breast feeding in Uganda is universal with 98% of children breastfed for at least sometime. EBF is not a traditional concept; beliefs and practices of early supplementation are common around the world. Supplementation with other liquids and foods occurs too early in Uganda and only 63% of Ugandan children under 6 months are exclusively breastfed (UDHS, 2002). The amount of EBF done in Uganda is over estimated by the Demographic Health Survey, the indicator used is EBF the day before the survey and not EBF since birth. EBF is known to protect against diarrhoea diseases, chest infections and other infections with the protective effect enhanced by greater exclusivity of the breastfeeding (WHO, 2002).

Although EBF is able to lower child mortality by 13% it is not being promoted on a large scale because of fear of HIV transmission through breast milk. The prevalence of HIV among pregnant women in Uganda has declined from an average of 30% in 1992 to an average of 8.7% in some urban areas and 4.2% in rural areas (ACP, 2000). The HIV epidemic has undermined efforts aimed at promotion of breastfeeding (Latham MC and Kisanga P, 2001). Furthermore, expensive approaches of dealing with HIV have removed the focus from breastfeeding as an inexpensive way of promoting optimal child health (Black et aI, 2003). Breastfeeding still seems to be a reasonable trade off for poor African HIV -positive mothers particularly if it is exclusive (Iliff et al , 2005). Another constraint to promotion of exclusive breastfeeding for six months, as recommended by WHO, is the uncertainty about its impact on the micronutrient status of infants (WHO, 2001).

Peer counselling is a proven cost effective approach for changing behavior, for example prevention of HIV infection (Hutton et aI, 2003). Several studies have also examined its impact on breastfeeding behavior (Shaw et aI, 1999; Morrow et aI, 1999; Haider et aI,

2000; Martens PI, 2002). More than half of the studies done so far have shown an effect of peer counselling on breastfeeding practices and no effect was reported by the other studies, however it is not known whether the same is true for African communities. This study intends to explore the promotion of EBF through trained peer counsellors, so as to combat poor feeding practices and as a result reduce infant morbidity and mortality in Africa.

2

***Literature review***

**Breastfeeding**

The benefits of breastfeeding and the negative effects of artificial feeding in underprivileged environments were clearly brought to public attention in the 1970s,backed by increasingly strong scientific support. As a result the International Code of Marketing of Breast-milk Substitutes and subsequent World Health Assembly Resolutions were created in the 1980s to avoid marketing of infant foods, teats and bottles in ways that could interfere with breastfeeding. In the early 1990s, UNICEF started the "Baby-friendly Hospital Initiative" (BFHI), which decreased many of the practices in the health care system that had a negative impact on breastfeeding. Together with many of the other child health programmes during these decades these interventions contributed to a reduction in child morbidity and child mortality. For infants, non-breastfeeding is dangerous and is associated with a high mortality rate in most of Africa. A WHO Study Team that estimated the odds ratio for dying if not breastfeeding to be 5.8 in the first two months of life for developing countries outside Africa but was unable to find a relevant data set for any African country (WHO, 2000).

**Exclusive breastfeeding**

Exclusive Breastfeeding (EBF) means that the infant receives nothing else other than breast milk, not even water. That breastfed children do not need anything besides breast milk, not even water, and not even in hot climates, was discovered in the 1970s(Almroth SG, 1978). A decade of research confirmed this, followed by an attempt to define breastfeeding into various patterns by WHO (WHO, 1991) and others (Labbock et al 1990). Since then, research has begun to suggest that, even in developed countries, EBF protects against gastrointestinal and respiratory infection, and that the protective effect is enhanced with greater exclusivity of the breastfeeding(WHO, 2002). Exclusivity also increases the duration of amenorrhoea, positive for maternal health and birth spacing(WHO, 2002).

EBF is not a traditional concept; beliefs and practices regarding a wide range of early supplements are common around the world, and often seem to reflect beliefs expressed by early physicians such as Galen (Greiner T. 1998). It is not widely understood by health care professionals, given the lack of information on it even in most basic paediatric training. Thus EBF is not widely practiced anywhere except perhaps in much of Scandinavia and parts of some countries in other regions, including a few African countries. The best source of data on the duration of exclusive breastfeeding in developing countries is the Demographic and Health Surveys run by Macro International Inc. and funded by USAID. In only 23% of the 61 national surveys from Sub-Saharan Africa available online is the median duration of exclusive breastfeeding longer than one month. Even this overestimates the amount of exclusive breastfeeding being done in Africa, as the indicator used is not "EBF since birth," but "EBF the day before the survey." So if EBF is able to lower child mortality by 13%, why is it not promoted at scale already today? There are two main reasons: fear of HIV transmission through breast milk and uncertainty about the micronutrient status in children exclusively breastfed for 6 months.

**EBF and risk of HIV transmission**

When an HIV -positive mother breastfeeds for 1.5 years or more, this appears to lead to an additional 15% risk of postnatal HIV transmission on average(John-Stewart et al, 2004) At a population level the epidemiological evidence is that exclusive breastfeeding should still be promoted even in countries with high prevalence of HIV(Coutsoudis et al, 2002). For many HIV positive mothers the risk of transmitting HIV to their children is outweighed by the risk of the infant dying if not breastfed because of the poor socioeconomic and environmental conditions they live in(Kuhn et al, 1997; Iliff et al, 2005) Limited evidence suggests that for these women and their children EBF is even more important, not just for minimising respiratory and gastrointestinal infections but also perhaps in reducing the risk of HIV transmission(Coutsoudis et al 2001). The HIV epidemic has undennined breastfeeding promotion efforts especially in countries that would benefit the greatest (Latham et ai, 2001) In addition, expensive approaches of dealing with HIV have removed the focus from breastfeeding as an inexpensive way of promoting optimal child health (Black RE et ai, 2003)

**EBF and micronutrient status**

Exclusively breast-fed babies seem to grow well for up to six months in both developed and developing countries (Kramer, 2002), suggesting that severe deficiency of most micronutrients is unlikely. However, in recently deciding to recommend EBF for six months(WHO, 2001), WHO warned .that, based on one randomized trial in Honduras(Dewey et al, 2001) infant iron status might not be optimal at six months and that data were inadequate to judge what effect six months of exclusive breastfeeding might have on infant status regarding certain other micronutrients, particularly zinc and vitamin A (WHO, 2001), (Butte et al, 2002), (Kramer et al 2003). That such basic research to establish the "normal" micronutrient status of infants exclusively breast-fed from birth has not been done reflects the fact that almost no researchers have utilized a strict definition of EBF and that very few infants in most countries have been fed that way. The use of a strict definition in doing such research would seem justified, as the gut environment, including its micro flora, is likely to be influenced by the addition of most types of supplements and even medications such as antibiotics.

**Peer Counselling**

The constraints discussed above have meant that efforts towards optimal promotion of EBF have been rudimentary so far, and in Africa have come almost to a complete

standstill in recent years. EBF promotion therefore needs to be reshaped in this era of HIV. The challenge is to urgently put back on track the promotion of exclusive breastfeeding for the benefit of the world's children without losing credibility by doing it indiscriminately. Peer counselling is a proven cost-effective approach in, changing behaviour, for example, preventing HIV infection (Hutton et al, 2003). Several studies have also examined its impact on breastfeeding behavior. Based largely on quasi-experimental studies, peer counselling can have an impact on the duration of any breastfeeding (Grummer-Strawn et al, 1997; Schafer et al, 1998; Gross et a, 1998; Shaw et al, 1999; Martens et al,2002). Kristin in the USA (Kristin et al, 1994) obtained increases in EBF among women who obtained services from peer counsellors compared to those for whom requested services were not available. In Bangladesh in a trial randomised by community(Haider et al, 2000) and) in Mexico, randomising at individual level also obtained substantial increases(Morrow et al, 2000. In Belarus found an increased duration and degree (exclusivity) of breastfeeding(Kramer et al 2001). However, in the USA two trials failed to have much impact(Caulfield et al, 1998), (McInnes et al, 2000). Green (Green CP, 1999) summarized the results of four unpublished quasi-experimental studies. Impacts of peer counselling in intervention compared to comparison/control groups were seen in studies done in Chile and Honduras. In another study in Honduras and one in Mexico, little effect was achieved, presumably due to limited implementation. Thus we feel that peer counselling is promising enough to be chosen as the intervention to focus on but not proven enough (indeed, hardly tested at all in Africa) to prevent it from being ethically randomized.

***Problem Statement***

More than 10 million children die each year from preventable causes and almost all from poor countries (Black RE et al, 2003). About 41 % of child deaths occur in sub-Saharan Africa from diarrhoea, pneumonia, malaria and HIV/AIDS; with under-nutrition as an underlying cause of two-fifths of all child deaths (UNICEF, 2003). The promotion of exclusive breastfeeding (EBF) is capable of lowering child deaths by 13% (Black RE et al, 2003). In Uganda only 63% of children under 6 months are exclusively breastfed (UDHS, 2001). The HIV epidemic has further undermined breastfeeding promotion efforts and expensive approaches of dealing with HIV have removed the focus from breastfeeding as an inexpensive way of promoting optimal child health. Lack of knowledge about the micronutrient status of exclusively breastfed infants also presents a barrier to promotion of EBF. Studies elsewhere suggest that peer counselling can often achieve dramatic increases in EBF rates. However the role of peer counselling in promotion of EBF has not been studied in an African setting.

***Justification of the Study***

About two-thirds of child deaths can be prevented by interventions that are available today and are feasible for implementation in low-income countries. EBF is one of the most effective child health interventions currently feasible for implementation at population level in low-income countries. It can lower infant mortality by 13%, and by an additional 2% were it not for the fact that breastfeeding transmits HIV. A second constraint to promotion of EBF for six months, as recommended by WHO, is uncertainty about its impact on the micronutrient status of infants. This study will utilise a peer counselling approach (randomised at community level) combined with the production of information that may in the longer run help overcome other constraints to EBF. It also seeks to test an upgraded version of EBF-promotion and to quantify its health benefits, cost-effectiveness and implications for the health care system. In particular, it will deal explicitly with each of the constraints to EBF, especially in Africa and ultimately giving back to Africa a powerful and cheap intervention to prevent child mortality and morbidity.

***Research Question***

Does individual peer-counselling given in the late ante-natal period and post-natally improve the prevalence of exclusive breastfeeding at 3 months in an African setting?

***Hypothesis***

**Null Hypothesis**

There is no association between peer counselling for exclusive breastfeeding and the prevalence of exclusive breastfeeding at 3months.

***Objectives***

**General Objective**

To assess the effect of peer counselling in increasing the prevalence of exclusive

breastfeeding at 3 month and strengthen the evidence base regarding the optimal duration for EBF.

**Specific objectives**

1. To determine the effect of peer counselling on EBF prevalence at 3 months.

2. To assess the effects of the trial on infant micronutrient status and growth measured by weight,length (WAZ, HAZ, HWZ) at 3, 6, 12 and 24 weeks of age.

3. To assess the effect of the trial on infant morbidity (2 weeks diarrhoeal disease prevalence up to 3 months).

4. To relate infant morbidity, growth (WAZ, HAZ, HWZ at 12 and 24 weeks) and

micronutrient status at 24 weeks to actual feeding pattern.

5. To assess the cost effectiveness of the intervention in terms of cost per averted

child morbidity, and per additional Disability Adjusted Life Years.

Conceptual Framework

Environmental factors

Family income

Source of water

Health services

Breastfeeding practices

Personal factors

Increase in rates of infants predominatly breastfed for longer duration (6 months)

Peer counselling

Cultural beliefs

**Reduction in infant morbidity and mortality**

***Methodology***

**Study Design**

This is a community intervention trial, randomized at community level with 12 clusters in each arm. Clusters will be made up of 1 or 2 villages with a population of at least 1 000 people. Care will be taken to allow for corridors in between clusters. Data will be collected using a standard questionnaire. The main outcome measure will be exclusive breastfeeding prevalence at 3 months.

**Study site**

The study will be conducted in Mbale district, eastern Uganda at the foothills of Mount Elgon and bordering Kenya. Mbale Municipality is the administrative centre of Mbale District as well as the regional centre for the Eastern Region. Mbale district is divided into 4 counties, the study will be conducted in Bungokho County (18 clusters) and in Mbale municipality (6 clusters). The clusters in municipality have been selected from all the three divisions namely Industrial Division ,Northern Division, and Wanale Division. Bungokho county surrounds Mbale municipality and consists of 11 sub counties. Clusters have been selected from Bukonde, Bukiende, Bungokho, Bungokho-Mutoto, Busiu, Busoba, Nakaloke and Namanyonyi, sub-counties . The population in Bungokho is predominantly Bagisu, the main language is Lumasaba and most of the people are peasant farmers.

***Participants***

**Target Population**

Pregnant mothers who opt to breastfeed their infants in Uganda.

**Accessible Population**

Pregnant mothers who opt to breastfeed their infants in Mbale district.

**Study Population**

Pregnant Mothers less than 40 years old, who opt to breastfeed their infants, resident in the study area and consent to participate in the study.

**Inclusion Criteria**

Mothers in their last trimester of pregnancy and intend to breastfeed, less than 40 years of age, resident in the selected clusters and consent to participate in the study.

**Exclusion criteria**

Mothers with intentions to move and infants born with serious diseases or deformities that prevent breastfeeding will be excluded from the study.

Consent

Informed consent will be obtained from participants prior to data collection. The consent form will be translated into the local language. Participants will not be named on the questionnaires or on any reports arising out of the study.

***Recruitment***

Each cluster will have a recruiter ("pregnancy monitor") who will be a mature woman of good repute living within the cluster. The pregnancy monitors will inform the research centre about pregnant women that may be eligible to participate in the study. A research assistant will be sent to the given cluster to ascertain eligibility, get consent to participate in the study and conduct the recruitment interview.

***The Intervention***

Peer counsellors will be identified and selected from the 12 clusters randomised to peer counselling. These will be women in the reproductive age group (25-45 years) and will be provided with approximately 20 hours of training. Approximately 20 peer counsellors will be trained (about double the number needed), to allow for attrition, sickness, etc. Peer counselling will be done during home visits (arranged at the convenience of the time of day) to the mother. Peer counsellors will provide substantial information, encouragement, skills and support for achieving exclusive breastfeeding, if possible for six months. Trained in listening, they will address specific concerns of mothers, correct misinformation, and do these without making mothers feel defensive or inadequate. They will be provided with motivational materials and will share these not only with mothers, but with other family members and anyone else who may have an influence on how mothers feed their babies. When necessary, they will provide referral to the lactation counsellor for any breastfeeding problems that may arise.

After the informed consent mothers assigned to peer counselling will be visited by the peer counselor on 1 occasion before delivery, at weekly intervals in the first 12 weeks post natally and fortnightly up to 24 weeks. The peer counselor will deliver messages on the advantages of EBF, provide simple technical information for overcoming constraints, provide referral of more complicated breast problems and provide reminders and encouragement to remain or return to exclusive breast feeding. Lactation managers will be available for all women included in the study.

***Randomization***

The clusters selected for randomization will be chosen in a way that each cluster represents a social and administrative unit. This will minimise interaction between (pregnant) women and mothers (of infants) in intervention and control clusters, and decrease the risk of contamination. Random allocation will be done by individuals not participating in the implementation of the trial. After the 24 clusters are identified, a list of relevant characteristics (such as population size, proximity to health facilities, roads, schools markets and trading centers) will be obtained. Clusters with similar characteristics will be paired and then randomized with the first of each matched pair to EBF-promotion or no intervention.

***Blinding***

Research assistants ( data collectors) and data analysts will be blinded to the random allocation of the clusters. Ascertainment of outcome will be done by research assistants not involved in the exposure allocation; these will be blinded as much as possible.

***Sample Size Calculations***

24 clusters (12 with intervention, 12 with no intervention):

We need 432 (~20) mother-infant pairs'in 12 clusters per arm (24 clusters in total)

yielding a total of 864 (~840) mother-infant pairs. On an average (and preferably without too large a spread) each cluster shall yield 36 deliveries over the 1 year recruitment period, i.e. 36*24=864 mothers-infant pairs, corresponding to 18*24=432 mothers-infant pairs each year.

With a 3% birth rate (30 births/lOOO inhabitants per year) and expecting that we can recruit to and keep in the study 75% of all pregnant women and the babies they give birth to, each cluster should yield an average (and without too large a spread) of 18/0.75=24 pregnancies per year.

***MEASUREMENTS***

**Baseline characteristics (Possible Confounding factors)**

Age of the mother, parity of the mother, mean length of education, occupation, average

length of EBF with previous children, measures of maternal morbidity, type and place of

delivery,marital status, household size, social economic status, use of clinical/medical services.

**Infant feeding practices**

The 24 hour recall of a list of food items, the weekly recall of a list of food items and the ever recall will be used to collect infant feeding practices at week 3, week 6, week 9, week 12 and at 24weeks.

**Anthropometric measurements**

Birth weight, and weight and length of infants at, 3 weeks and 12weeks; Weight and height of mother at 3weeks post partum.

**Morbidity and mortality**

Morbidity (passive surveillance) for diarrhoea, ARI and hospitalisation episodes and mortality through the verbal autopsy.

**Micro-nutrient status**

Blood specimens (About 4mls from each child) will be collected from all the 840

children at 24 weeks. Analysis would be performed on a subset depending on resources and the rest of the samples would be stored for future analysis. The following tests; Ferritin, CRP, Copper-Zinc, Serum retinal will be performed.

**Outcome measures**

Primary outcome measure will be the **EBF prevalence at 3 months**.

Secondary outcome:

- Decrease in diarrhoea
- Improved growth
- Micronutrient status

***Data collection***

The research team will consist of the principal investigators, 20 peer counsellors, 24

pregnancy monitors and 8 research assistants. A data collection tool for handheld computers (EpiHandy) backed up by paper questionnaires will be used. Questionnaires designed on a desktop PC (using EpiHandy - Survey Manager) will be downloaded to handheld computers. The interviewers will enter the data directly on the handheld computer by clicking on the touch sensitive screen. There is an instant check for validity, which does not allow certain types of erroneous responses to be entered. Using a handheld GPS, connected to the handheld the geographical position of each interview is automatically recorded.

All mothers will be interviewed at recruitment to establish eligibility and get informed consent, and to obtain data on possible confounding factors as well as descriptive statistics. They will be interviewed again at 3, 6, 12 and 24 weeks after birth to determine feeding patterns, morbidity data and survival. Anthropometric measurements will be taken at 3weeks, 12 weeks and 24 week interview. Mothers' weights and heights will be taken at the 3 week interview.

Infant feeding patterns will be established through food frequency recalls. The 24 hour recall of breast milk and a list of food items that could have been introduced to the baby with frequency (no. of feeds per day). Weekly food recall with average number of times breast fed or given a food item (from a list of food items). Ever recall for a list of food items including the first time a particular item was introduced. Blood specimens will be collected from all the 840 children at the 24 week interview. About 4mls of blood will be drawn from each child for the following tests; Ferritin, CRP, Copper-Zinc, Serum retinal. Analysis would be performed on a subset depending on resources and the rest of the samples would be stored for future analysis.

The morbidity data will be collected through repeated cross sectional surveys. Diarrhoea and ARl will be asked for through 24-hour recalls, 1 week recalls and 2 week recalls. Data will be collected on hospitalisation for malaria, diarrhoea and ARl. Verbal autopsies (with or without a death certificate) will be performed for the mothers and babies who pass away during the study period.

***Data Management***

Collected data will be temporarily stored in a secure and encrypted database on a memory card on the handheld and automatically uploaded to the site-office database on a daily basis. A data management centre will be located in South Africa at an established research centre. The data from the site-office will be transferred (based on secure technologies) on weekly basis to the data management centre to minimise the risk of data loss. From this centre anonymous datasets will be made available to the principal investigators for further analysis.

The data stored on the handheld computer will be encrypted and protected with a strong password and username combination. The site office will be secured with appropriate measures to prevent theft and unwanted access to hardware and data. Access to collected data at the site will be strictly controlled by the onsite data manager. This security will be possible through the use of industry standard SQL databases i.e. MS SQL Server or MSDE (Microsoft Desktop Engine) that have high level of reliability and built-in security. Strict backup procedures at site level and at the data management centre will include daily backup to appropriate media which will be safely stored. The weekly transfer of data from sites to the data management centre will be an additional backup method.

***Data analysis***

Data will be transferred from the handheld to the PC and will be analyzed using Stata version 8.0. Continuous variables will be summarized into means, median, and standard deviation. Categorical variables will be summarized into proportions. The results will be presented in form of tables and figures. Relative Risks, Attributable Risk Reduction, and Number Needed to Treat (counsel) will be estimated. Micronutrient data will be analysed in three ways: (1) intent to treat, to see what impact the intervention had, (2) according to feeding patterns to examine the effects when non-compliance is taken into account, and (3) to obtain data on infants exclusively breast-fed since birth for periods longer than four months. This will help to establish a norm or baseline regarding how infant micronutrient status changes over time during exclusive breastfeeding, what kind of variations occur, and whether determinants of this variation (especially low levels) can be identified.

***Quality control***

Formative research on infant feeding patterns has been conducted and analysed using qualitative research methods to better understand local perceptions and factors that lead people to provide early supplementation. This knowledge will be important in the adaptation of the training for the peer counsellors and lactation managers. This research also includes issues like "Who could be an effective peer counsellor?", "How would the peer counsellors work?" and "What are the key messages needed?" The peer counsellors will be recruited from the selected clusters randomised to peer counselling and will be trained. The peer counselor trainers have attended a training the WHO breastfeeding course.

The research assistants will be recruited from the study area and will be able to speak the local language. They will under-go training on the study objectives, use of the handheld computers and anthropometry. Steps will be taken to avoid visits by the peer counselor coinciding with visits of the research assistants.

The questionnaires will be translated into Lumasaba and back translated into English. The food recall interview to be done at the 3, 6, and 12 week after child birth will be validated with the one week food recall recommended by WHO prior to the implementation of the study to test for sensitivity and reproducibility. The questionnaires will be pre-tested as part of the validation study. Standard operating procedures (SOPs) will be developed for the implementation of the study, training of research assistants and peer counselors; data collection, management and analysis.

Two research assistants sufficiently skilled (clinical officer/ laboratory technologist) will be trained to perform the phlebotomy on each child. Blood will be collected In heparinised vacutainers, protected from sunlight, centrifuged, and frozen locally in a freezer with a back-up generator until all samples have been taken. Then they will be transported frozen to a central laboratory, where analysis will be done for plasma retinol, zinc, ferritin, and C-reactive protein (to protect against falsely low retinol and high ferritin values due to infection or inflammation).

The weighing scales and height boards will be zeroed before each field visit and before each measurement is taken. They will be checked for accuracy every week with known standard weights. The height and weight measures are those recommended by UNICEF and WHO.

A data manager will be trained in management of the database, systems used and support to the interviewers using the handheld computer. Using handheld computers the data will be checked for consistency and validated at the time of entry. Standard operating procedures (SOP's) will be developed for each stage of data collection, handling and analysis. These will include data validation, methods for data cleaning and quality control. Support will be ensured through using various communication channels, including online systems, between the technical advisors / site coordinators and field staff., This will enable ongoing capacity development of field staff and problems with hardware or software to be dealt with rapidly and thereby avoiding data loss.

***Ethical Issues***

Institutional permission to conduct the study will be obtained from the Faculty of Medicine, Makerere University; Uganda National Council of Science and Technology; Ministry of Health and Mbale Local Government. Individual written consent will be obtained from the respondents in the study. The consent process will explain the nature of the study, the risks and benefits of participating in the study, the intervention and that allocation is by a random process. Confidentiality of information and the right of the respondent to withdraw from the study at any time during the study will be explained to the participants. Research assistants will be trained on confidentiality.

***Limitations***

This being a behavioral change intervention it is difficult to blind the mothers and the investigators to the allocation of the intervention.

***Dissemination of Results***

Relevant staff will participate in discussing and drawing conclusions from the completed data analyses. Internal guidelines for authorship have already been agreed upon. Key staff will submit and present abstracts to relevant national, regional and international scientific and policy-making meetings. The consortium will disseminate the results through abstracts in meeting proceedings, scientific publications in international refereed journals, and prepared presentations for relevant authorities and policy makers.

1. Amendment of the PROMISE-EBF protocol to include prevalence of malaria as one of the outcome measures

- Introduction

Uganda, like many other African countries, has high malaria transmission rates, with 93% of the total population being at risk. Although all four species of malaria parasites exist in Uganda, Plasmodium falciparum is responsible for over 95% of cases. Malaria remains the major contributor to the disease burden in Uganda, being responsible for 39% of all outpatient visits and 35% of the inpatient visits.

Breastfeeding has been found to provide strong protection against infectious diseases. However, there is hardly any literature on the impact of breastfeeding on the development of malaria among infants. In a Bamako study, children breastfed for at least 2 years were found to have a lower risk of severe malaria. This was attributed to immunoprotective mechanisms, or alternatively, related to the fact that lactoferrin, a protein found in breast milk and neutrophils, with antibacterial properties as well as lipoprotein remnants inhibit the invasion of hepatocytes by malarial sporozoites, because they compete for the same receptor in the hepatocyte plasma membrane.

We therefore wish to assess the impact of exclusive breastfeeding on malaria prevalence among 24 week infants. Furthermore, we will evaluate the effect of using trained peer supporters for exclusive breastfeeding on malaria prevalence among infants.

- Objective

The main objective is to assess the effect of peer counseling on infant morbidity (prevalence of malaria at 24 weeks)

Materials and methods (remain the same as the approved study)

- Study design

A community randomized trial

Study Site

Mbale district, in Eastern Uganda. This district is divided into 4 counties, and the study will done in Bungokho county and Mbale municipality. The population of Bungokho is predominantly Bagisu and the people are mainly peasant farmers.

- Study Population

All infants aged 24 weeks within the study area.

- Sample size

For this malaria outcome, using Open Epi, a sample size of 368 infants with 184 in each arm will be sufficient to demonstrate a 30% decrease in prevalence of malaria, with a 95% confidence (1-alpha) and power of 80 considering prevalence rates for malaria parasitaemia (asymptomatic) among young children of 50% to 80% in Uganda

| Two-sided significance level(1-alpha): | 95 |  |
| --- | --- | --- |
| Power(1-beta, % chance of detecting): | 80 |  |
| Ratio of sample size, Unexposed/Exposed: | 1 |  |
| Percent of Unexposed with Outcome: | 50 |  |
| Percent of Exposed with Outcome: | 35 |  |
| Odds Ratio: | 0.54 |  |
| Risk/Prevalence Ratio: | 0.7 |  |
| Risk/Prevalence difference: | -15 |  |
|  | | |
| Sample size- Intervention group 184 | | |
| Sample size -Control group 184 | | |
| Total sample size 368 | | |

- Intervention

Promotion of Exclusive breastfeeding for the first six months of life

- Outcome measures

Prevalence of malaria at 6 months: In the intervention group receiving peer counseling, we expect a 15% reduction in the point prevalence of malaria in comparison to the control.

- Analysis: In this sub study, data will be analyzed in two ways,

(1) Intent to treat, to see what impact the intervention had,

(2) According to feeding patterns to examine the effects when non-compliance is taken into account,
